# Néoplasie endocrinienne multiple type 1: à propos d'un cas

**DOI:** 10.11604/pamj.2019.33.238.18053

**Published:** 2019-07-19

**Authors:** Gladys Anguezomo, Ghizlane El Mghari, Nawal El Ansari

**Affiliations:** 1 Service d'Endocrinologie, Diabétologie et Maladies Métaboliques, CHU Mohamed VI Marrakech, Maroc; 2 Faculté de Médecine et de Pharmacie de Marrakech, Université Cadi Ayyad, Marrakech, Maroc

**Keywords:** Multiple endocrine neoplasia type 1 (MEN1), pituitary adenoma, hyperparathyroidism, pNET, Néoplasie endocrinienne multiple type1 (NEM1), adénome hypophysaire, hyperparathyroïdie, pNET

## Abstract

La néoplasie endocrinienne multiple type1 (NEM1) est une maladie rare, définie par l'atteinte tumorale, chez le même sujet, d'au moins deux glandes endocrines affectant l'antéhypophyse, les parathyroïdes et le tissu endocrine duodéno-pancréatique. Cette pathologie héréditaire autosomique dominante est liée à la mutation du gène NEM1 codant pour la ménine et situé sur le chromosome 11q13. Il existe, toutefois, des formes sporadiques dans 8 à 14% des cas. La lésion endocrinienne initiale peut être unique dans environ 75%. Cependant, chacune des principales atteintes peut être inaugurale. Le cas rapporté ici est révélé par un adénome hypophysaire somatoprolactinique ayant un caractère agressif, ne répondant pas au traitement conventionnel. La découverte d'une hyperparathyroïdie primaire ainsi qu'une tumeur neuroendocrine du pancréas (pNET) sept ans plus tard en font toute la particularité. L'attitude thérapeutique est discutée au sein d'une équipe pluridisciplinaire spécialisée dans le domaine de la pathologie endocrine.

## Abstract (en)

Multiple endocrine neoplasia type 1 (MEN1) is a rare disease, defined as a tumor developing in at least two endocrine glands including the anterior pituitary gland, the parathyroid glands and the duodenopancreatic endocrine tissue. This disorder, inherited in an autosomal dominant pattern, is caused by mutations in the MEN1 gene encoding the tumor suppressor menin and located on chromosome 11q13. However, sporadic cases account for 8-14%. The first endocrine lesion may be solitary in approximately 75% of cases. However, all major alterations can be inaugural. We here report a case of multiple endocrine neoplasia type 1 revealed by aggressive somatoprolactinic pituitary adenoma which didn't respond to conventional treatment. The detection of primary hyperparathyroidism as well as neuroendocrine tumor of the pancreas seven years later make this a very particular case. Therapeutic options are discussed within the multidisciplinary team specialized in endocrine diseases.

## Introduction

La néoplasie endocrinienne multiple type 1 (NEM1 (ou syndrome de Wermer)) est une maladie génétique rare, caractérisée par l'association chez le même patient ou sujets apparentés, d'atteintes tumorales d'au moins deux glandes endocrines, incluant une hyperparathyroïdie, une tumeur endocrine du pancréas et un adénome hypophysaire. D'autres atteintes sont possibles mais moins fréquentes notamment des tumeurs endocrines corticosurrénales fonctionnelles ou non, bronchiques, thymiques ou gastriques. Des proliférations non endocrines pourraient aussi être rencontrées, lipome sous-cutané et angiofibrome cutané [1]. Il s'agit d'une maladie héréditaire autosomique dominante déterminée par des mutations du gêne, référencé MEN1 qui code pour la Ménine et situé sur le chromosome 11q13. Mais une forme non familiale ou sporadique peut être rencontrée chez 8 à 14% des cas avec NEM1 [2].

## Patient et observation

**Figure 1:**
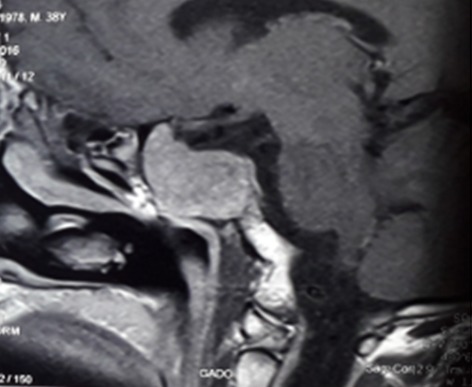
IRM hypophysaire montrant un macroadénome hypophysaire

**Figure 2:**
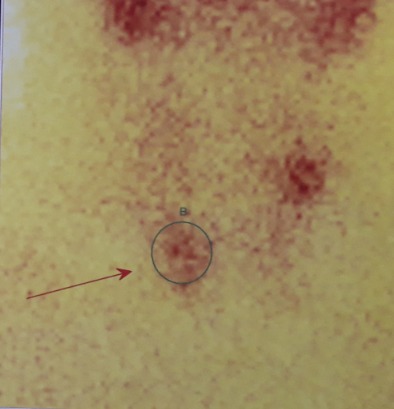
Image scintigraphique d'un adénome parathyroïdien

**Figure 3:**
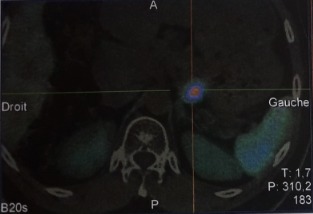
Image scintigraphique d'une tumeur endocrine du pancréas

**Figure 4:**
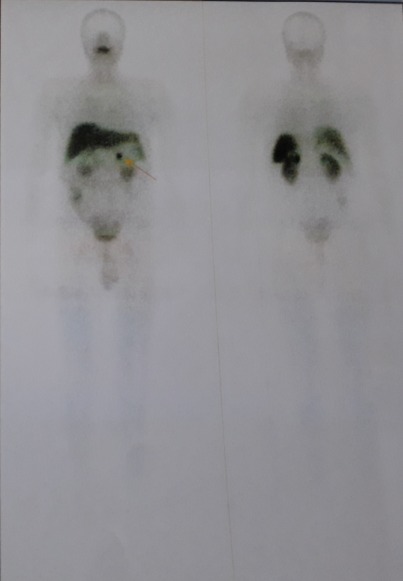
Image scintigraphique d'une tumeur endocrine du pancréas

Il s'agit d'un patient âgé de 39 ans ; ayant un antécédent de chirurgie hypophysaire avec un cas similaire chez la mère, opérée pour tumeur cérébrale non étiquetée. Présente une récidive d'un macroadénome hypophysaire révélé par un syndrome tumoral fait de céphalées en casque et troubles visuels d'installation progressive, des signes en faveur d'un déficit thyréotrope et gonadotrope. Le patient rapporte par ailleurs, des coliques néphrétiques et lithiase rénale. L'examen clinique révèle un syndrome dysmorphique avec faciès acromégaloïde notamment des rides épaissies, extrémités boudinées. Une galactorrhée à la pression, des OGE (Organes Génitaux Externes) d'aspect normal et lésions cutanées à type de neavus, une obésité grade 1. L'exploration hormonale a mis en évidence une hyperprolactinémie à 3633 ng/ml (157N), l'IGF1 (Insulin-like Growth Factor) élevée à 318 ng/ml était le seul élément en faveur de l'acromégalie, le dosage de la GH (Growth Factor ou hormone de croissance) sous HGPO (hyperglycémie provoquée par voie orale) étant revenu normal. Aussi un déficit hypophysaire multiple notamment thyréotrope et gonadotrope. Sur le plan radiologique, l'IRM hypophysaire objective une récidive de macroadénome hypophysaire compressif localement infiltrant, mesurant 40x31x20mm versus 20x44x27mm, avec engainement des sinus caverneux et compression des nerfs optiques (Figure 1). En outre, une atteinte opto-chiasmatique plus marquée du côté gauche au champ visuel. Le bilan de NEM 1 a montré, dans un premier temps, une hyperparathyroïdie primaire révélée par une hypercalcémie à 126 mg/l (corrigée à 127 mg/L), hypophosphérémie et une parathormonémie élevée à 268,1 pg/ml. La Scintigraphie au sesta-méthoxy-isobutyl-isonitride (MIBI) a mis en évidence la présence au niveau du pôle inférieur du lobe thyroïdien droit (LTD) et polaire gauche d'une rétention focalisée de méthoxy-isobutyl-isonitride (MIBI) en faveur de tissu parathyroïdien pathologique (Figure 2). Par ailleurs, une échographie cervicale: nodules thyroïdiens lobaire droit mesurant 8x4 mm et lobaire gauche mesurant 22x10 mm, classés Thyroid imaging reporting data system (TIRADS) 4A. Un prurit généralisé ainsi qu'un bilan biologique perturbé en l'occurrence un syndrome de cholestase avec phosphatases alcalines (PAL) à 7N et Gamma glutamyl-transpeptidase (GGT) à 49N, ainsi qu´un syndrome de cytolyse hépatique, ont motivé la réalisation d'une Bili-IRM qui a objectivé un processus lésionnel de la tête du pancréas, de 1,5*3cm, infiltrant la lame rétro-portale et engainant le bas cholédoque, avec dilatation des voie biliaire intra-hépatique (VBIH) et extra-hépatique (EH), lésion du corps du pancréas de 1,5*1,4cm évoquant une tumeur neuroendocrine et kyste de la queue du pancréas. Le dosage de la chromogranine A, revenu positif a également permis de conforter le diagnostic d'une tumeur neuroendocrine du pancréas. Une scintigraphie des récepteurs de la somatostatine (Octréoscan^®^) est donc réalisée dans le cadre du bilan d'extension. Mis dans un foyer d'hyperfixation isolé correspondant à une masse tissulaire du corps du pancréas, compatible avec une origine neuro-endocrine (Figure 3, Figure 4). La masse céphalique du pancréas n'a pas de traduction scintigraphique, origine autre que neuro-endocrine ainsi qu'une absence d'anomalie de fixation viscérale ou osseuse qui soit en faveur de localisation secondaire. La prise en charge de ce patient, consistera dans un premier temps à une chirurgie notamment d'une thyroïdectomie totale avec parathyroïdectomie totale; cholécystectomie avec biopsie pancréatique et une dérivation cholédoco-duodénale. L'examen anatomo-pathologique montre un aspect histologique compatible avec adénome parathyroïdien, un parenchyme pancréatique dystrophique, une tumeur neuroendocrine du pancréas grade 2. Pour l´adénome hypophysaire, une radiochirurgie fractionnée a été indiquée devant le caractère agressif de la tumeur avec maintien de la cabergoline à forte dose (6cp par semaine) et mise sous somatuline Lp 120mg à raison d´une injection par mois.

## Discussion

Un patient est considéré comme ayant une NEM1, s'il présente deux des trois principales tumeurs liées au gêne MEN1 affectant les parathyroïdes, les îlots pancréatiques et l'antéhypophyse. La prévalence de la NEM1 dans la population générale reste estimée à 1/20000 et 1/40000 [2,3]. Au moment du diagnostic, la lésion endocrinienne initiale est unique dans 75% des cas, chacune des principales atteintes parathyroïdienne, hypophysaire et pancréatique peut être inaugurale. Toutefois, l'atteinte des parathyroïdes reste la première manifestation de la maladie chez la majorité des patients et la plus fréquente à 95% des cas, suivie des tumeurs neuroendocrines du pancréas (pNET) (50%) puis les adénomes hypophysaires (40%) [4,5]. Le mode révélation chez notre patient a été l'adénome hypophysaire, suivi des autres principales atteintes respectivement parathyroïdienne et pancréatique. Chez les patients NEM, les adénomes hypophysaires sont présents dans 40% des cas. En général, ces adénomes hypophysaires surviennent à un âge plus jeune, soit avant 45 ans dans 75% des cas et sont le plus souvent des macroadénomes ayant une agressivité particulière et une sécrétion plurihormonale [6-8]. Le caractère agressif du macroadénome somatoprolactinique chez notre patient, par l'existence, d'un envahissement des sinus caverneux englobant les artères carotidiennes et compression des nerfs optiques, de récidive par la progression du volume tumoral de plus de 30% et l'absence de normalisation de la prolactinémie avec résistance aux thérapies modales, concordant avec les marqueurs cliniques d'agressivité des adénomes hypophysaires.

L'hyperparathyroïdie (HPT) primaire constitue le trouble initial du syndrome chez la majorité des patients (90-100%), sans que cela soit obligatoire [2,5]. L'atteinte parathyroïdienne a été la deuxième manifestation de la maladie chez notre patient, découverte dans le cadre du bilan de NEM sept ans après le diagnostic de l'adénome hypophysaire. Dans plus de deux tiers des cas, il s'agit d'une hyperplasie diffuse des parathyroïdes volontiers asymétriques et dans un tiers des cas d'adénomes parathyroïdiens, pouvant être multiples dans 16% des cas de NEM1. Le carcinome parathyroïdien rapporté chez les patients avec NEM1 mais reste rare soit 0,28% de tous les sujets présentant une NEM [5,9]. La tumeur neuroendocrine du pancréas a constitué la dernière manifestation de la maladie chez notre patient. Cette tumeur classée T1NoMo (grade 2) selon la classification TNM de l'European Neuroendocine Tumor society (ENET) et l'OMS 2010. Le taux de chromogranine A étant également en faveur. Les tumeurs neuroendocrines du duodéno-pancréatiques observées dans la NEM1 sont non fonctionnelles pour la plupart. L'insulinome est la tumeur la plus sécrétante (8%) mais le gastrinome reste fréquente (40 %) dans un contexte de NEM1. Les glucagonomes et VIPomes (6-10%) sont moins fréquents au cours de le NEM1 mais ont un potentiel de malignité supérieur. Les patients avec NEM1 ont une espérance de vie réduite avec un taux de mortalité de 50% avant l'âge de 50 ans. Le pronostic de ces patients pourrait être amélioré par la détection présymptomatique et traitement spécifique des tumeurs [10,11].

## Conclusion

La NEM1 est un syndrome héréditaire rare qu'il faut savoir évoquer devant l'association de différentes atteintes, notamment hypophysaire, parathyroïdiennes et du tissu endocrine duodéno-pancréatique chez un individu donné ou dans une famille. Le pronostic est principalement lié au risque métastatique des tumeurs pancréatiques et thymiques, et aux complications liées aux diverses hypersécrétions hormonales. La prise en charge des tumeurs neuroendocrines de la région duodénopancréatique est plus difficile quand ces tumeurs s'intègrent dans le cadre d'une NEM1. L'attitude thérapeutique chirurgicale est discutée par des équipes spécialisées dans le domaine de la pathologie endocrine.

## Conflits d’intérêts

Les auteurs ne déclarent aucun conflit d'intérêts.
